# The hookworm *Ancylostoma ceylanicum* intestinal transcriptome provides a platform for selecting drug and vaccine candidates

**DOI:** 10.1186/s13071-016-1795-8

**Published:** 2016-09-27

**Authors:** Junfei Wei, Ashish Damania, Xin Gao, Zhuyun Liu, Rojelio Mejia, Makedonka Mitreva, Ulrich Strych, Maria Elena Bottazzi, Peter J. Hotez, Bin Zhan

**Affiliations:** 1 Sabin Vaccine Institute and Texas Children’s Hospital Center for Vaccine Development, National School of Tropical Medicine, Baylor College of Medicine, Houston, TX 77030 USA; 2 McDonnell Genome Institute, Washington University School of Medicine, St. Louis, MO 63108 USA; 3 Division of Infectious Diseases, Department of Medicine, Washington University School of Medicine, St. Louis, MO 63108 USA; 4 Department of Biology, Baylor University, Waco, TX 76706 USA

**Keywords:** Hookworm, *Ancylostoma ceylanicum*, Intestine, Transcriptome, Vaccine candidate

## Abstract

**Background:**

The intestine of hookworms contains enzymes and proteins involved in the blood-feeding process of the parasite and is therefore a promising source of possible vaccine antigens. One such antigen, the hemoglobin-digesting intestinal aspartic protease known as *Na-*APR-1 from the human hookworm *Necator americanus*, is currently a lead candidate antigen in clinical trials, as is *Na-*GST-1 a heme-detoxifying glutathione S-transferase.

**Methods:**

In order to discover additional hookworm vaccine antigens, messenger RNA was obtained from the intestine of male hookworms, *Ancylostoma ceylanicum*, maintained in hamsters. RNA-seq was performed using Illumina high-throughput sequencing technology. The genes expressed in the hookworm intestine were compared with those expressed in the whole worm and those genes overexpressed in the parasite intestine transcriptome were further analyzed.

**Results:**

Among the lead transcripts identified were genes encoding for proteolytic enzymes including an *A. ceylanicum* APR-1, but the most common proteases were cysteine-, serine-, and metallo-proteases. Also in abundance were specific transporters of key breakdown metabolites, including amino acids, glucose, lipids, ions and water; detoxifying and heme-binding glutathione S-transferases; a family of cysteine-rich/antigen 5/pathogenesis-related 1 proteins (CAP) previously found in high abundance in parasitic nematodes; C-type lectins; and heat shock proteins. These candidates will be ranked for downstream antigen target selection based on key criteria including abundance, uniqueness in the parasite *versus* the vertebrate host, as well as solubility and yield of expression.

**Conclusion:**

The intestinal transcriptome of *A. ceylanicum* provides useful information for the identification of proteins involved in the blood-feeding process, representing a first step towards a reverse vaccinology approach to a human hookworm vaccine.

**Electronic supplementary material:**

The online version of this article (doi:10.1186/s13071-016-1795-8) contains supplementary material, which is available to authorized users.

## Background

Human hookworm infection remains one of the leading neglected tropical diseases (NTDs), affecting more than 400 million people living in developing countries and causing the loss of about 3.2 million disability adjusted life years (DALYs) [1, 2]. Hookworm is a blood-feeding nematode that hooks onto the host intestinal mucosa using sharp teeth or cutting plates, causing significant host blood loss. As a consequence, hookworm infection is the major cause of iron-deficiency anemia in endemic regions [3]. Current control of hookworm infection mainly relies on mass drug administration with a single annual dose of an anthelmintic such as albendazole or mebendazole. However, a recent systematic analysis revealed that anthelmintic treatment with mebendazole had no impact on the improvement of anemia in hookworm-infected regions [4] and is associated with a low cure rate overall [5, 6], while the cure rates with albendazole were highly variable [7]. Therefore, development of a vaccine has emerged as a practical and feasible alternative technology to control hookworm infection or complement anthelmintic drug treatment [1, 8]. A human hookworm vaccine is considered both cost-effective and cost-saving relative to mass drug administration [9].

Hookworm infection begins when infective larvae penetrate the skin of the host and migrate through the circulatory system and the lungs before reaching the intestine, where they develop into adult worms and start blood-feeding [3]. The hookworm’s survival exclusively depends on blood-feeding and the digestion of blood proteins including hemoglobin and serum proteins as a major source of nutrition [10]. During the past decade, the molecular basis of this blood-feeding process and the digestion has been partially identified as a cascade of hemoglobinases [11]. After the worm takes up blood into the intestine, the red blood cells are lysed by hemolysin and hemoglobin is released. The released hemoglobin is degraded and digested by a series of hemoglobinases, initiating cleavage of the hemoglobin molecule by an aspartic protease (APR) [12, 13], followed by further digestion with several cysteine proteases [10, 14] and metalloproteinases [15]. These proteases are expressed in the brush border membrane of the parasite's intestine [10, 15, 16]. A vaccine development strategy is in process that focuses on interfering with the blood-feeding process of the hookworm. It targets proteins, including enzymes, found in the gut of the major human hookworm, *Necator americanus*, and involved in the degradation of hemoglobin and the detoxification of breakdown products of the process [1]. Vaccination elicits anti-enzyme antibodies that can reach the hookworm intestine through blood feeding and subsequently inactivate their target enzymes in the hookworm’s digestive tract and other organs. Among the lead candidate antigens are *Na-*APR-1, a cathepsin-D aspartic protease required for hemoglobin digestion and *Na-*GST-1, a glutathione-S transferase involved in detoxification of toxic heme derived from hemoglobin digestion – both molecules induce significant protective immunity in vaccinated animals against hookworm infection [16, 17]. These two antigens have been selected as the leading hookworm vaccine antigens for product development and are currently in phase 1 clinical trials [1, 8]. *Ac*-CP-2, another cysteine protease of the canine hookworm *Ancylostoma caninum*, was also determined to be expressed on the intestine brush-border membrane. Like *Na-*APR-1, laboratory animals vaccinated with recombinant *Ac*-CP-2 also produced antibodies that stunted worm development and reduced egg count after being challenged with hookworm larvae [18]. In addition to their efficacy in preclinical studies, a second rationale for choosing hookworm gut antigens is that antigens from the hookworm intestine are not directly exposed to the host immune system during natural infection, thereby reducing the likelihood of eliciting host IgE responses and allergic antibodies. This issue had previously thwarted other vaccine development efforts for infective larval stage-derived antigens [19].

In an effort to identify additional antigens in the hookworm intestine that might be suitable for the development of novel vaccine antigens and therapeutics, we undertook an antigen discovery program. Based on the success of previous reverse vaccinology programs for selecting bacterial vaccine antigens [20], we are interested in initiating a similar approach for the human hookworm vaccine based on the gut transcriptome. Such antigens could be added to, or considered alternatives to, the current candidates, depending on the progression of the clinical trials. We believe that the complete profile of the hookworm intestine’s gene expression pattern could significantly facilitate this selection process. For this study we used *Ancylostoma ceylanicum* as a model, a hookworm that infects both animals (e.g. dogs, hamsters) and humans (in focal areas of Malaysia and elsewhere in Southeast Asia) and expresses proteins sharing high homology with the two major human hookworms *N. americanus* and *A. duodenale* [21] which only infect humans and cannot properly infect laboratory animals without the use of immunosuppressant [22]. The full transcriptome of the *A. ceylanicum* adult intestine was sequenced and analyzed. The results reveal some new macromolecules likely involved in parasite survival in the mammalian host with the potential to serve as future vaccine candidates or drug targets for controlling hookworm infections.

## Methods

### Preparation of *A. ceylanicum* intestinal RNA

Hookworm *A. ceylanicum* was maintained in hamsters according to protocol AN-5762 approved by Baylor College of Medicine’s Institutional Animal Care and Use Committee (IACUC). Fresh *A. ceylanicum* adult worms were recovered from the intestines of hamsters 21 days after infection with *A. ceylanicum* larvae [23]. In order to avoid contamination from the worm’s reproductive organs and eggs, only male worms were collected. Total hookworm intestinal tissue was isolated from 50 male adult worms. The anterior esophagus and any contaminating reproductive organs were removed. The isolated intestines were preserved immediately in RNA*later* (Qiagen, Hilden, Germany) to prevent degradation of RNA. Total RNA was extracted from the hookworm intestines using Trizol reagent (Invitrogen, Carlsbad, CA, USA), then digested with Ambion Turbo DNase (Ambion/Applied Biosystems, Austin, TX, USA) to remove any contaminated chromosomal DNA. The quality and yield of the purified RNA were assessed using an Agilent 2100 bioanalyzer (Agilent Technologies, Cedar Creek, TX, USA) using the ratio of 28S/18S rRNA and a NanoDrop ND-1000 spectrophotometer (NanoDrop Technologies, Wilmington, DE, USA). The integrity of the purified RNA was determined using the RNA Integrity Number [24].

### RNA-seq library construction and Illumina sequencing

Messenger RNA (mRNA) was prepared from the purified total RNA using the MicroPoly(A)Purist™ Kit (Ambion/Applied Biosystems, Austin, TX, USA), and then converted to cDNA using Ovation®’s RNA*-*Seq V2 kit (NuGen Technologies, Inc., San Carlos, CA, USA) with both a poly-A and a random primer. An Illumina sequencing library was constructed according to the manufacturer's instructions (Illumina Inc., San Diego, CA, USA). Briefly, 1 μg cDNA was sheared using a Covaris S220 DNA Sonicator (Covaris Inc., Woburn, MA, USA) and fragments with a size range of 200–400 bp were collected. Single Primer Isothermal Amplification (SPIA) adapters were added to those fragments, followed by PCR amplification to enrich adaptor-ligated fragments. Quantitative PCR (Kapa Biosystems Inc., Woburn, MA, USA) was used to determine the concentration of the resulting library and to produce the cluster counts appropriate for the Illumina platform. The library was finally loaded into the flow cells of the HiSeq2000 sequencer to generate paired end reads of approximately 100 bp in length [21].

The mRNA*-*sequence reads were processed using an in-house *Perl* script to remove adaptor sequences as well as those with low complexity or derived from non-target sources (golden hamster genome database, GCF_000349665.1, and GenBank Bacterial Sequences database, GBBCT). The processed reads were mapped to the unpublished *A. ceylanicum* genome (available at http: nematode.net [25]), using Tophat 2.0.8 [26] with the parameter mate-inner-distance set to 300. Breadth of coverage and read count of individual *A. ceylanicum* gene transcripts were calculated using Refcov, version 0.3 (http://gmt.genome.wustl.edu/packages/refcov/) [27] and HTSeq-count (http://www-huber.embl.de/users/anders/HTSeq/doc/count.html) [28], respectively. Genes with at least 50 % breadth of coverage were considered to be expressed in the male *A. ceylanicum* intestine. The corresponding counts were used to represent the expression abundances of those genes. The significantly abundant gene transcripts in *A. ceylanicum* intestine were identified with the Fisher’s exact test in Bioconductor package *edgeR* on the basis of comparison of the gene transcript counts in the intestinal transcriptome with the whole male worm transcriptome. A corrected false discovery rate (FDR) cutoff of 0.05 was used.

### Quantitative reverse transcription PCR (qRT-PCR)

To validate the results from the RNA-seq experiments, five genes with higher expression levels in the intestine than in the whole worm were chosen for quantitative reverse transcription PCR (qRT-PCR). Total RNA was freshly extracted from whole worms and isolated intestines of *A. ceylanicum*. Total cDNA was reverse-transcribed using the SuperScript III First-Strand Synthesis System per the manufacturer’s protocol (Invitrogen, Waltham, USA). Quantitative PCR was performed using a PowerUp™ SYBR® Green (Life Technologies, Carlsbad, USA) cocktail in a ViiA7 real-time PCR detection system (Applied Biosystems). The PCR conditions were 95 °C for 1 min, followed by 35 cycles of 95 °C for 25 s, 55 °C for 20 s, 72 °C for 1 min, and concluded by a 10 min incubation step at 72 °C. The 18S rRNA of *A. ceylanicum* (DQ464371.1) was selected as a standard comparison control gene.

### Bioinformatics analysis

The functional annotations based on primary sequence level was done using Interproscan (https://www.ebi.ac.uk/interpro/), and further classified into different Gene Ontologies (GO). We further added various sub-categories within each GO-ontology and weighted them with FPKM (Fragments Per Kilobase of transcript per Million mapped reads, a measure of the relative expression of an individual transcript) values for each gene. Those transcripts highly expressed in the worm’s intestine were determined through comparison with data from the whole adult male transcripts available from the SRA for *A. ceylanicum* (accession numbers: SRX1116908 and SRX1116915) downloaded and employed in this study. The Merops database (https://merops.sanger.ac.uk/), a manually curated peptidase and inhibitor database, was used to annotate and classify peptidases in order to identify which proteases or peptidases are highly expressed in the hookworm intestine. Pepunit.lib sequences from release 9.12 were downloaded from the website (http://merops.sanger.ac.uk/download/) and used to create a custom Blastp database using BLAST command line tools and an e-value of less than 10^-6^. The hits were weighted by using their FPKM values and then summed to plot using proportional values. Molecules involved in transmembrane transport were identified using the TrSSP dataset (http://bioinfo.noble.org/TrSSP/?dowhat=Datasets). The transporter sequences were downloaded from the Transporter Substrate Specificity Prediction Server (TrSSP) and used for BlastP searches against the hookworm intestinal *RNA-Seq* genes obtained in this study by setting an e-value of less than 10^-6^. Phylogenetic trees were generated for those functional protein homologues identified in this study using Phylogeny.fr [29] (http://www.phylogeny.fr/index.cgi). Signal peptides were predicted using Phobius Server (http://phobius.sbc.su.se/) [30]. The transmembrane domains were predicted using TMHMM (http://www.cbs.dtu.dk/services/TMHMM/) [31].

## Results and discussion

### Isolation of hookworm intestines

**Fig. 1:**
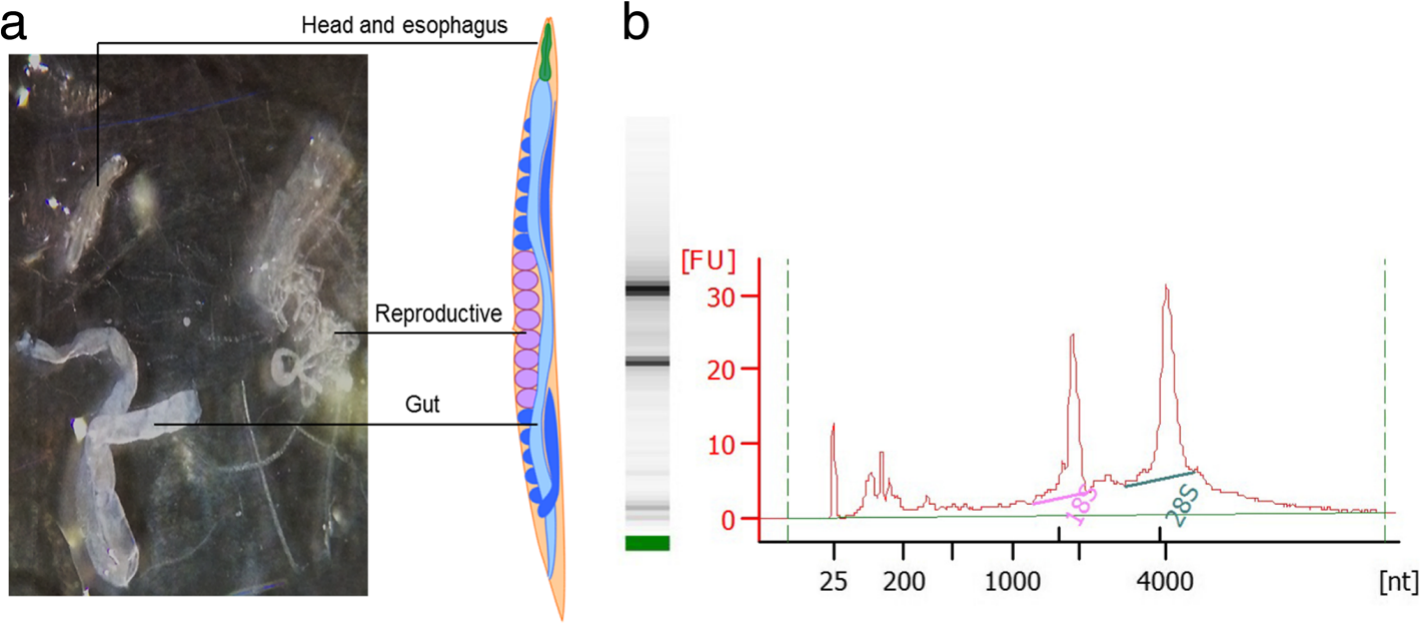
The intestines were isolated from *A. ceylanicum* male worms avoiding esophagus and reproductive organs (**a**), and total RNA was extracted and treated with DNase. The integrity of the total intestinal RNA was analyzed by electrophoresis and the RNA Integrity Number (RIN) for the purified RNA was 8.6 (**b**)

The intestines of hookworms were isolated from living adult male *A. ceylanicum* worms collected from infected hamsters under microscopy (Fig. 1a). Total RNA was extracted from the isolated intestine and the total RIN for the hookworm intestinal RNA was 8.6 on a scale that extends from 10 (intact) to 2 (degraded), indicating that most of the RNA had remained intact throughout the purification process (Fig. 1b).

### Hookworm intestinal transcriptome analysis

The RNA-seq library constructed using the sample obtained above was subjected to Illumina RNA sequencing. The RNA-seq data generated from the adult male intestine of *A. ceylanicum* is available from NCBI under BioProject ID PRJNA72583 with SRA accession number of SRX1127457. The transcriptional profile analysis revealed that a total of 8,495 genes, versus a total of 15,892 transcripts in the whole genome (BioProject ID PRJNA72583), had been expressed in our sample of the hookworm *A. ceylanicum* intestine. Among the 8,495 *A. ceylanicum* intestinal genes, 6,559 have predicted IPR domains according to the InterPro scan analysis. A total of 7.1 % of intestinal transcripts possessed a signal peptide that is statistically higher than that in the whole worm (4.1 %) (Chi-square test, *χ* ^2^ = 104.988, *P*-value < 0.0001), suggesting that more transcripts in intestine encode proteins that secret into the intestine. Hookworm intestine contained 19.0 % of transcripts that contain transmembrane domain, which is similar to that in the whole worm (19.8 %). Secreted or membrane-associated proteins are expected to be involved in the worm’s digestive process. Comparison of the FPKM value for the intestine expressed transcripts with the corresponding gene FPKM-derived from the transcriptome of the whole *A. ceylanicum* male worm, resulting in 964 genes being expressed at significantly higher level in the *A. ceylanicum* intestine than in the whole worm based on the Fisher’s exact test in Bioconductor package edgeR [32] (Table 1).

**Table 1:** Summary of the *A. ceylanicum* adult intestinal transcriptome and characterization compared to the whole worm

	Intestine	Whole worm
No. of genes	8,495	15,892
Total FPKM (Intestine)	1,221,742	1,348,733
Average length	369 nt	320 nt
No. of determined functions	6,559	not available
No. of unknown functions/hypothetical proteins:	1,936	not available
Genes abundantly expressed in intestine	964	
Genes encoding signal peptides	607 (7.1 %)*	650 (4.1 %)
Genes encoding transmembrane domains	1,614 (19.0 %)	3,154 (19.8 %)

*P < 0.001 compared to whole worm

### Genes highly expressed in the hookworm intestine

**Fig. 2:**
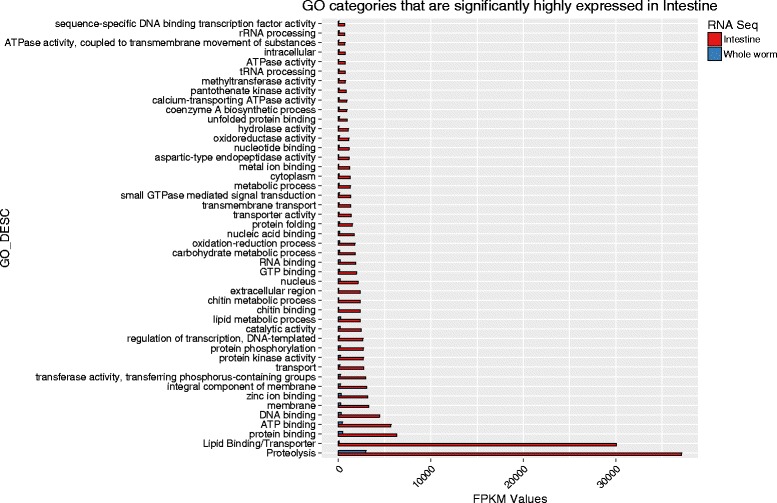
Gene ontologies of those genes expressed predominantly in the intestine *versus* the whole adult worm, expressed as FPKM values

The genes highly expressed in the hookworm intestine are likely involved in blood-feeding and therefore constitute important targets for hookworm vaccine or therapeutic drug development [11]. The 100 genes with the highest FPKM in the intestine are listed in Additional file 1: Table S1, together with a putative functions or characterization. The higher expression levels in the intestine for five selected genes were also confirmed by qRT-PCR using independently prepared batches of RNA samples from whole worms and intestines of *A. ceylanicum*, with 12.0–68.8 % increase in intestine compared to the whole worm calculated by 2^(-ΔΔCt)^ (Table 2). After comparison of the relative transcript abundance to the whole worm, a gene ontology analysis was carried out on those transcripts highly expressed in the intestine. The analysis determined that those proteins with hydrolytic functions or binding/transporting activities are the most highly expressed genes in the intestine compared to the whole worm, especially those involved in protein digestion and the transport of digested macromolecules such as amino acids, fatty acid, sugar and ions. The top ten most abundant groups of genes expressed in intestine belong to the categories of proteolysis [FPKM intestine/whole worm (ratio) = 37,125/2,971 (12.5)], lipid-binding/transporters [30,077/78 (385.6)], protein-binding [6,317/476 (13.3)], ATP-binding [5,681/435 (13.1)], DNA-binding [4,456/276 (16.1)], membrane [3,289/232 (14.2)], zinc ion-binding [3,163/289 (11)], integral component of membrane [3,061/219 (14)], transferase activity [2,959/212 (14)], other transporters [2,732/164 (16.7)] (Fig. 2). These most abundantly expressed genes and their categorization are also shown in Additional file 1: Table S1.

**Table 2:** qRT-PCR confirmation of higher transcript levels of selected genes in intestine than in the whole worm presented by 2^(-ΔΔCt)^, the percentage change in intestine compared to the whole worm

Gene ID	Description	FPKM (intestine)	FPKM (whole worm)	qRT-PCR change in intestine/whole worm 2^(-ΔΔCt)^
ANCCEY_ 10351	Nematode specific protein with unknown function	19853	1270	68.8 %
ANCCEY_ 05890	hypothetical protein NECAME_13614	27188	3676	64.0 %
ANCCEY_ 09250	Nematode specific protein with unknown function	12540	1265	18.2 %
ANCCEY_ 07095	intestinal serine protease	8137	553	16.1 %
ANCCEY_05930	von Willebrand factor and C-type lectin domain	20518	278	12.0 %

We further explored various subcategories within the main gene ontologies and weighted those using FPKM values. Those protein categories with the highest expression levels in the intestine are analyzed below.

#### Proteases

With a total FPKM of 37,125 in the intestine, compared to 2,971 FPKM in the whole worm, proteases constitute the most highly expressed proteins in the worm’s intestinal transcriptome. The Merops database was used to run BLASTP on 8,495 protein sequences from RNA*-*seq using an e-value of less than 10^-6^. We obtained 3,473 hits, of which 674 were unique. We determined that 518 of those unique hits were classified as peptidases and 156 of them as peptidase inhibitors.

**Fig. 3:**
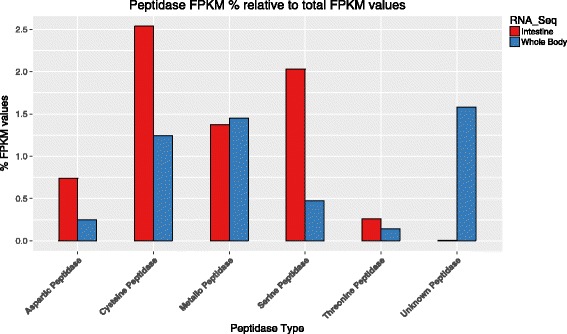
Expression levels of various peptidase classes in the intestine of adult *A. ceylanicum* compared to those in the whole worm

When peptidases were further sub-classified into aspartic peptidases, cysteine peptidases, metallopeptidases, serine peptidases, threonine peptidases and unknown peptidases, we found that the most common peptidase in the intestine were the cysteine peptidases (2.6 % of the total FPKM), followed by serine peptidases (2.1 %), metallopeptidase (1.4 %), aspartic peptidases (0.7 %) and threonine peptidases (0.2 %). Compared to the whole worm transcripts, the cysteine peptidase, serine peptidase, aspartic peptidase and threonine peptidase were preferentially expressed in the intestine (Fig. 3), indicating their possible involvement in blood digestion.

Searching the intestinal protease profile identified in this study, the aspartic peptidase ANCCEY_13850 shares 89 % amino acid sequence identity with *Na-*APR-1. Its expression level is high in the intestine, with the ninth highest FPKM level observed in the protease category (Table 3, FPKM 579.3). Moreover, a cysteine protease, ANCCEY_09304, was identified that shares 55.0 % sequence identity with *Ac*-CP-2 (Table 3). In addition to the homologues of *Na-*APR-1 or *Ac*-CP-2 in the *A. ceylanicum* intestine involved in the hemoglobin digestion, there are additional proteases that may contribute to the proteolysis of hemoglobin or other serum proteins adapted to the blood feeding of hookworm. There were a total of 26 proteases, including serine proteases, cysteine proteases, aspartic proteases, metalloproteases, and aminopeptidases, which were expressed with an at least 8 times higher FPKM in the intestine than in the adult worm except for ANCCEY_13850 with 1.5 times higher (Table 3). These proteases could be the important targets for therapeutic or preventive vaccine or drug development.

**Table 3:** Proteases highly expressed in intestine with FPKM at least 8 times higher than those in whole worm except for ANCCEY_13850 (1.5 times higher)

Gene ID	Description	FPKM (intestine)	FPKM (whole worm)	FPKM ratio intestine/whole
ANCCEY_07095	serine-type peptidase	8137	553	14.7
ANCCEY_09301	cysteine-type peptidase	2654	342	7.8
ANCCEY_07093	serine-type peptidase	2520	188	13.4
ANCCEY_07094	serine-type peptidase	1769	120	14.8
ANCCEY_00957	aspartic-type protease	1087	37	29.1
ANCCEY_12936	cysteine-type peptidase	976	93	10.5
ANCCEY_07096	serine-type peptidase	899	93	9.7
ANCCEY_10060	cysteine-type peptidase	700	72	9.7
ANCCEY_13850	aspartic-type protease^a^	579	376	1.5
ANCCEY_10673	catabolic protease	386	48	8.0
ANCCEY_09304	cysteine-type peptidase^b^	204	9	22.7
ANCCEY_02985	zinc-metalloprotease	127	0.2	636.0
ANCCEY_03546	aminopeptidase N family	112	7	16.9
ANCCEY_15589	aminopeptidase N family	101	9	11.0
ANCCEY_03485	serine-type protease	89	5	16.5
ANCCEY_02980	zinc-metalloprotease	86	0	86.3
ANCCEY_08904	cysteine-type proteinase	77	9	8.5
ANCCEY_06121	aspartic-type protease	53	5	11.5
ANCCEY_02983	zinc-metalloprotease	46	0	45.6
ANCCEY_01806	cysteine-type peptidase	25	1	27.4
ANCCEY_07150	serine-type protease	24	1	16.9
ANCCEY_09045	Prolyl oligopeptidase family	18	0	17.8
ANCCEY_09192	aspartic-type protease	15	0.2	75.0
ANCCEY_02981	Zinc-metalloprotease	14	0.1	144.0
ANCCEY_09053	prolyl oligopeptidase family	14	0	14.0
ANCCEY_04091	peptidase family M13	11	0	11.0

^a^89 % identity with *Na-*APR-1, ^b^55 % identity with *Ac*-CP-2

#### Transporters

**Fig. 4:**
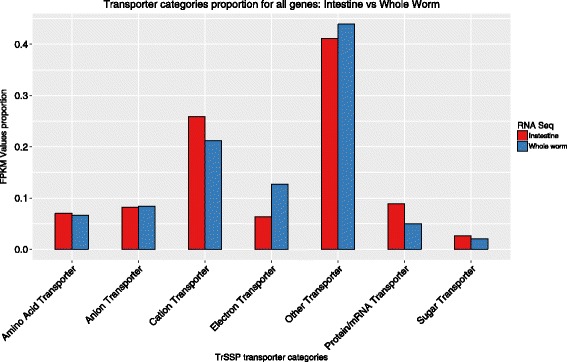
TrSPP transporter categories: FPKM relative proportions for genes significantly expressed in the intestine of adult *A. ceylanicum* compared to the whole worm

The hookworm intestine expresses different transporters with functions related to the absorption of digested macromolecules and the maintenance of homeostasis between the worm and outside environment [21, 33, 34]. The specific binding proteins, transporters or pumps on the gut membrane are necessary for the uptake of metabolites such amino acids, glucose, lipids, ions and water after blood feeding digestions [35]. A search against the TrSSP database revealed that virtually all of the categories of transporters are expressed in the intestine at much higher levels than in the whole worm. These highly expressed transporters include amino acid transporters, protein/mRNA transporters, sugar transporters, ion transporters and other transporters. The latter two categories of transporters (cation and other) may indicate their importance in ion exchange and other material transport, such as for lipids, ATP, or other nucleotides (Fig. 4). Amino acids and small peptides derived from blood likely play a key role in hookworm nutrition. However, their uptake and absorption rely on the amino acids/peptide transporters produced by hookworm intestine. Deletion of an intestinal peptide transporter from free-living nematode *Caenorhabditis elegans* reduced the uptake of digested peptides from the gut lumen, resulting in delayed development, reduced progeny and body size [36].

In addition to the high expression of amino acid/peptide transporters, the genes involved in lipid-binding and transportation are also highly expressed in the hookworm gut (Figs. 2 and 4). Vitellogenin and fatty acid and retinol-binding (FAR) proteins represent two major categories of lipid-binding proteins involved in cross-membrane transport. More than three vitellogenins (ANCCEY_09758, ANCCEY_15699, ANCCEY_01522) are exclusively expressed in the hookworm gut with high FPKM from 4298.3–7970.4 (Additional file 1: Table S1; Table 4). Vitellogenin is a lipid transport and storage protein consisting of four domains including lipovitellin-1, phosvitin, lipovitellin-2, and a von Willebrand factor type D domain (vWFD) [37, 38]. The 3-D structure of vitellogenin reveals that phosvitin and lipovitellin domains form amphipathic structures of a lipid pocket to receive their lipid cargo [39]. The vWFD is located at the cysteine-rich C-terminus of vitellogenin with homology to human von Willebrand factor type D domains. Unlike human vWFD though, metazoan vWFDs do not contain RGD motifs, which are associated with extracellular matrix binding. The conservation of cysteine-rich vWFDs throughout the metazoan, including nematodes, implicates functional importance, possibly protecting vitellogenin from being digested by proteases [39]. The knockdown of vitellogenin in *C. elegans* had previously been shown to result in reduced survival after bacterial infection, indicating the crucial role of vitellogenin in nematode’s life-cycle [40].

**Table 4:** Lipid binding proteins highly expressed in the intestine of *A. ceylanicum*

Gene ID	Description	FPKM (intestine)	FPKM (whole worm)	FPKM ratio Intestine/whole
ANCCEY_09758	Lipid transport protein/vitellogenin/vWF	7970	0	7970.0
ANCCEY_15699	Lipid transport protein/vitellogenin/vWF	6008	3	2002.7
ANCCEY_01522	Lipid transport protein/vitellogenin/vWF	4298	0	4298.0
ANCCEY_03501	(SRPBCC) ligand-binding domain superfamily	4261	0	4261.0
ANCCEY_04371	Nematode fatty acid retinoid binding protein	411	41	10.0
ANCCEY_00853	Nematode fatty acid retinoid binding protein	384	5	76.8
ANCCEY_04363	BPI/LBP/CETP N-terminal domain	351	10	35.0
ANCCEY_04372	Nematode fatty acid retinoid binding protein	133	16	8.3

Another category of highly expressed lipid-binding proteins identified in this study includes fatty acid and retinol-binding proteins (FAR). Lipids and retinoids are relatively insoluble in water and, in their oxidative free form, can be toxic to membranes. Consequently, parasitic nematodes secrete a structurally unique class of FAR proteins to sequester fatty acids and retinoids from the host necessary for the worms’ essential biological processes [41–43]. Crystallization of *Na-*FAR-1 reveals its large and more complex internal ligand-binding cavity for binding to lipid [43]. In this study, we have identified at least three FAR proteins (ANCCEY_04371, ANCCEY_00853 and ANCCEY_04372) with high FPKM values (132.7–411.2).

#### Glutathione-S transferases (GSTs)

**Fig. 5:**
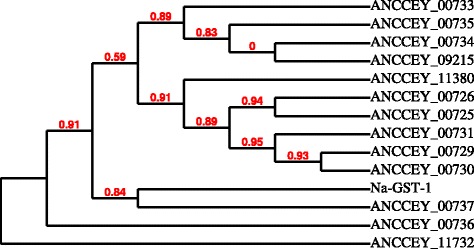
Phylogenetic tree comparing *Na-*GST-1 with 13 newly identified GSTs expressed in the *A. ceylanicum* intestine, with branch support values in red. ANCCEY_00737 shares the highest similarity with *Na-*GST-1 (66 % amino acid identity)

After digestion of hemoglobin, free heme is released into the hookworm intestine. Due to its oxidative iron, free heme is a potent enzyme inhibitor and source of reactive oxygen species that may damage the parasite’s structure and DNA [44]. As in other blood-feeding parasites such as *Plasmodium* spp. and *Schistosoma* spp. [35, 45, 46], hookworm has evolved mechanisms to detoxify the free heme by expressing nematode-specific Nu-class GSTs that have higher affinity heme-binding sites through homodimerization of two GST molecules [47–49]. Due to the importance of hookworm GSTs involved in the heme binding and detoxification, vaccination with hookworm GSTs produced protective immunity in laboratory animals against hookworm larval challenge [17, 47, 48], and thus *Na*-GST-1 from *N. americanus* has been selected as the leading vaccine candidate for human hookworm infection [48]. To date, three GSTs have been cloned from *N. americanus* adult worms. Here a total of 13 new GSTs were identified in the transcriptome of the *A. ceylanicum* adult intestine (Fig. 5). ANCCEY_00737 shares the highest level of similarity with *Na-*GST-1 (60.0 % amino acid identity). Possibly some of them are involved in the detoxification of heme and carrying of reduced non-toxic heme to functional sites required by this parasite, because heme is a co-factor of some essential enzymes and hookworm is considered as heme auxotrophic [50]; therefore hookworm gut-expressed GSTs could be candidates for vaccine or drug development.

#### ASP/CAP proteins

Upon stimulation by a host signal during penetration into the skin, arrested hookworm infective larvae assume feeding and development, and simultaneously secrete many proteins related to migration and parasitism in the host. Some of those activated secreted proteins are called *Ancylostoma*-secreted proteins or activation-associated secreted proteins (ASPs) [51, 52]. This group of proteins exists in a wide range of eukaryotic organisms, including plants, vertebrates and invertebrates including helminths, and belongs to the large family of cysteine-rich secretory proteins, antigen 5, and pathogenesis-related 1 proteins (CAPs) [53], also known as sperm-coating protein (SCP)-like extracellular proteins, or SCP/Tpx-1/Ag5/PR-1/Sc7 (SCP/TAPS) proteins [54]. Except for their association with establishment of parasitism in the host, the exact function of ASPs remains mostly unknown. Recent studies have demonstrated that *Na-*ASP-2 of the human hookworm *N. americanus* binds to the human B-cell antigen receptor complex CD79A and downregulated about 1,000 B-cell messenger RNAs including factors involved in leukocyte transendothelial migration pathways and the B-cell signaling receptor pathway, indicating nematode-secreted ASP/CAP proteins may cause immunomodulation as a strategy of immune evasion [55]. Vaccination with recombinant ASP-1 or ASP-2 secreted by hookworm larvae produced significant protective immunity in different animal models [17, 23, 56]. Unfortunately, the larvae-secreted ASPs induced an IgE response during natural infection and pre-existing anti-*Na-*ASP-2 IgE caused a significant allergic response upon vaccination with recombinant *Na-*ASP-2, which has stopped the development of *Na-*ASP-2 as a vaccine in endemic areas [19]. However, it has been shown that ASP’s fusion to human IgG Fc fragment could significantly reduce the anti-*Na-*ASP-2 IgE-triggered histamine release [57].

**Fig. 6:**
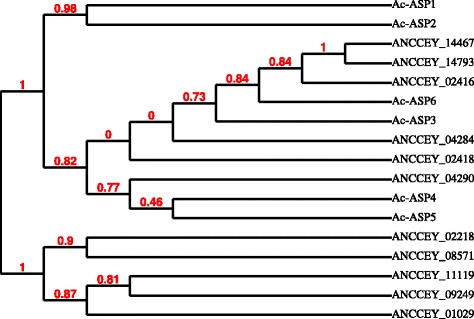
Phylogenetic tree comparing the eleven most abundant ASPs expressed in the adult *A. ceylanicum* intestine with ASPs previously identified in *A. caninum* adults (*Ac*-ASP-3, -4, -5 and -6) and larvae (*Ac*-ASP-1 and *Ac*-ASP-2), with branch support values in red

The *A. ceylanicum* genome contains a total of 432 ASP genes, more than any other hookworm species described so far [21, 34, 58, 59]. Except for those secreted by activated larvae, some well-studied ASPs are only expressed in adult worms [60]. In this study, we identified 62 ASP genes expressed in the hookworm intestine, of which 11 are within the 100 most abundant gene products in the intestine, second only to the protease group (Table 5; Additional file 1: Table S1). Phylogenetic analysis suggests that these ASPs are closely related to the adult worm expressed proteins, *Ac*-ASP-3, *Ac*-ASP-4, *Ac*-ASP-5 and *Ac*-ASP-6 [60], but less related to the larvae-secreted *Ac*-ASP-1 [51] and *Ac*-ASP-2 [52] (Fig. 6). Due to their specific expression in the adult intestine, these proteins are not expected to be exposed to the host immune system, and therefore there is less concern regarding the induction of an IgE response during natural infection, possibly making them suitable vaccine antigen candidates.

**Table 5:** ASP/CAP proteins highly expressed in *A. ceylanicum* adult intestine

Gene ID	Description	FPKM (intestine)	FPKM (whole worm)	FPKM ratio intestine/whole
ANCCEY_04290	one PR-domain ASP	3140	422	7.4
ANCCEY_02416	two PR-domains ASP	2583	232	11.1
ANCCEY_02418	one PR-domain ASP	2523	179	14.1
ANCCEY_01029	one PR-domain ASP	1676	130	12.9
ANCCEY_08571	two PR-domains ASP	1375	87	15.8
ANCCEY_02218	one PR-domain ASP	1304	87	15.0
ANCCEY_14467	one PR-domain ASP	1165	14	83.2
ANCCEY_11119	two PR-domains ASP	917	92	10.0
ANCCEY_09249	one PR-domain ASP	823	2	411.5
ANCCEY_14793	one PR-domain ASP	568	0	568.0
ANCCEY_04284	one PR-domain ASP	446	0	446.0

#### C-type lectins

C-type lectins are sugar-binding proteins mediating both pathogen recognition and cell-cell interactions using structurally related Ca^++^-dependent carbohydrate-recognition domains (C-type CRDs) [61]. Nematodes secrete certain CTLs homologous to some key receptors of the mammalian host immune system, such as CD23 and CD206 [62–64]. It is conceivable that nematode-secreted CTL-like products interfere with the immunological recognition or effector function, suggesting a potential role at the host-parasite interface, possibly as a strategy to evade the host immune response [65]. In *C. elegans* more than 278 CTL-like genes have been found [66], with some of them induced upon bacterial infection [67]. The *A. ceylanicum* intestine also expresses an abundant number of C-type lectins. In this study 8 CTLs were identified among the 100 most abundant proteins in the *A. ceylanicum* intestine (Table 6). Two of them (ANCCEY_05930 and ANCCEY_10784) contain a von Willebrand factor type A domain at their N-terminus, which might make these proteins important in adhesion processes involving platelets, collagen and other proteins. This is especially true for ANCCEY_05930 which is extremely highly abundant in the hookworm intestine (FPKM 20518.0).

**Table 6:** C-type lectin proteins highly expressed in the *A. ceylanicum* adult intestine

Gene ID	Description	FPKM (intestine)	FPKM (whole worm)	FPKM ratio intestine/whole
ANCCEY_05930	C-type lectin + vWF	20518	278	73.8
ANCCEY_14101	C-type lectin	9784	99	98.8
ANCCEY_13406	C-type lectin	7763	375	20.7
ANCCEY_00895	C-type lectin	5931	268	22.1
ANCCEY_10709	C-type lectin	1453	0	1453.0
ANCCEY_12199	C-type lectin	450	0.1	450.0
ANCCEY_00717	C-type lectin	364	0.4	910.0
ANCCEY_10784	C-type lectin + vWF	225	27	8.3

#### Heat shock proteins (HSPs)

HSPs are a family of highly conserved proteins induced upon different kinds of environmental stress such as heat, infection, inflammation, toxins, starvation, hypoxia, pH extremes, and nutrient deprivation [68]. Not only acting as chaperones by stabilizing intracellular proteins to maintain correct folding or by helping those proteins damaged to refold, HSPs also play significant roles in immunomodulation such as antigen presentation, activation of lymphocytes and maturation of dendritic cells [69]. Due to their important roles in parasite survival, HSPs have been chosen as vaccine candidates and the protective efficacy of HSP vaccinations has been shown against various parasitic infections including *Plasmodium yoelii* [70], *Brugia malayi* [71], *Leishmania donovani* [72] and *Trichinella spiralis* [73]. In this study, we identified 8 HSPs within the 100 most abundant proteins expressed in hookworm intestine, including HSP20, 40 and 70 (Table 7). The high expression in intestine and conserved functions for worm’s survival suggest these hookworm intestine-expressed HSPs could be the targets for preventive vaccines or pharmaceutical drugs.

**Table 7:** Heat shock proteins among the top 100 most abundant proteins expressed in the *A. ceylanicum* adult intestine

Gene ID	Description	FPKM (intestine)	FPKM (whole worm)	FPKM ratio intestine/whole
ANCCEY_00293	HSP20	3121	5	624.2
ANCCEY_01927	HSP20	2500	2	1250.0
ANCCEY_01990	HSP20	2202	0	2202.0
ANCCEY_01992	HSP20	1851	0	1851.0
ANCCEY_05462	HSP20	721	5	144.2
ANCCEY_04125	HSP70	492	44	11.2
ANCCEY_05040	HSP40	415	61	6.8
ANCCEY_10608	HSP40	387	41	9.4

## Conclusions

Whereas reverse vaccinology approaches have yielded promising results in terms of identifying vaccine targets against bacterial pathogens, so far this approach has had limited success for more complex eukaryotic organisms. Among the reasons why anthelmintic vaccines have not benefited from reverse vaccinology approaches include the large genome of parasitic helminths and difficulties in shaping moderate to high throughput approaches for protein expression and preclinical testing in laboratory animal models. To address the first component, we have narrowed our antigen selection program for the human hookworm vaccine to the parasite’s gut transcriptome.

The hookworm intestine expresses a wide range of proteins or enzymes involved in blood-feeding and homeostasis between the parasite and its host. The functional antigens expressed in the hookworm gut have become major targets for vaccine and drug development. In order to discover additional hookworm vaccine antigens, the *A. ceylanicum* intestinal transcriptome was generated and analyzed. *Ancylostoma ceylanicum* was selected because of its relative convenience for vaccine testing in a hamster laboratory animal model. Analysis of the full transcriptome of the adult male *A. ceylanicum* intestine reveals that two categories of proteins were highly expressed in the hookworm intestine, namely proteolytic enzymes involved in blood digestion and transporter proteins involved in the absorption of nutrient metabolites and the maintenance of homeostasis between the parasite and its environment. Glutathione S-transferases involved in the binding and detoxification of oxidative heme derived from blood-feeding were also highly expressed in the intestine. In addition, other proteins including a family of cysteine-rich/antigen 5/pathogenesis-related proteins (CAP); C-type lectins; and heat shock proteins all also upregulated in the hookworm intestine and possibly contribute to the survival of worm in the host. Thus at least two classes of potential hookworm antigens are related to adult blood-feeding, whereas the function of the CAP proteins, C-type lectins, and heat shock proteins in the host-parasite relationship are still under investigation. More hookworm-specific intestinal proteins involved in blood-feeding may further be identified by comparing other available intestinal transcriptomes of nematodes such as *Ascaris suum* (non-blood-feeding) [74], *Trichuris suis* (tissue-feeding) and *Haemonchus contortus* (blood-feeding) [75]. In subsequent studies, the vaccine candidates identified here will be ranked for downstream antigen target selection based on criteria including abundance, possible functions related to survival, uniqueness in the parasite *versus* the vertebrate host, and ultimately solubility and yield of expression.

## Additional file

Additional file 1: Table S1. The 100 most abundant proteins in *A. ceylanicum* intestine. (PDF 326 kb)

## Abbreviations

APR: Aspartic protease
ASP: *Ancylostoma*-secreted protein
CAP: Cysteine-rich/antigen 5/pathogenesis-related 1 proteins
CP: Cysteine protease
CTL: C-type lectin
DALY: Disability adjusted life years
FAR: Fatty acid and retinol-binding protein
FDR: False discovery rate
FPKM: Fragments per kilobase of transcript per million mapped reads
GO: Gene ontologies
GST: Glutathione-S transferase
HSP: Heat shock protein
IACUC: Institutional animal care and use committee
NTD: Neglected tropical disease
SPIA: Single primer isothermal amplification
TrSSP: Transporter substrate specificity prediction server
vWFD: von Willebrand Factor type D domain
