# Supplementary material for: The hookworm Ancylostoma ceylanicum intestinal transcriptome provides a platform for selecting drug and vaccine candidates
Source: Parasit Vectors. 2016 Sep 27;9:518. doi: 10.1186/s13071-016-1795-8 (PMC5039805; doi:10.1186/s13071-016-1795-8)
Supplement: Additional file 1: Table S1. — The 100 most abundant proteins in A. ceylanicum intestine. (PDF 326 kb) [file 13071_2016_1795_MOESM1_ESM.pdf]

Additional file 1. Table S1 Top 100 most abundant proteins in *A. ceylanicum* intestine

Gene ID	FPKM intestine	FPKM ♂ worm	Size(kDa)	Predicted function domain
ANCCEY_05890	27188	3676	23.6	hypothetical protein NECAME_13614
ANCCEY_05930	20518	278	27.1	von Willebrand factor and C-type lectin domain*
ANCCEY_10351	19853	1270	24.2	Nematode specific protein with unknown function
ANCCEY_09250	12540	1265	8.5	Nematode specific protein with unknown function
ANCCEY_14101	9784	99	16.2	von Willebrand factor type A domain protein + CTL*
ANCCEY_15840	9243	0	25.8	Unknown hookworm specific protein
ANCCEY_07095	8137	553	25.3	intestinal prolyl carboxypeptidase (serine protease)
ANCCEY_09758	7970	0	138.4	Lipid transport protein/von Willebrand factor domain*
ANCCEY_13406	7763	375	13.9	C-type lectin domain containing protein*
ANCCEY_09592	7484	820	24.6	tenascin-C [*Danio rerio*]
ANCCEY_01889	6089	503	28.8	parasitic stage specific protein 1
ANCCEY_15699	6008	3	17.0	vitellogenin 1 [*Angiostrongylus vasorum*]
ANCCEY_00895	5930	268	17.1	von Willebrand factor and C-type lectin domain*
ANCCEY_01522	4298	0	58.4	vitellogenin [*Tanichthys albonubes*]
ANCCEY_03501	4261	0	24.9	Lipid-binding START domain
ANCCEY_04290	3140	422	32.1	SCP / Tpx-1 / Ag5 / PR-1
ANCCEY_00293	3121	5	18.3	HSP20
ANCCEY_00466	3071	1	20.8	hypothetical unknown nematode protein
ANCCEY_05068	3046	0	15.2	Protein of unknown function (DUF4106)
ANCCEY_11355	2902	210	32.3	hypothetical hookworm protein unknown
ANCCEY_00465	2715	18	29.1	hypothetical unknown nematode protein
ANCCEY_09301	2654	342	26.4	cathepsin B, cysteine protease*
ANCCEY_02416	2583	232	42.2	two-domain SCP / Tpx-1 / Ag5 / PR-1
ANCCEY_02418	2523	179	19.5	SCP / Tpx-1 / Ag5 / PR-1
ANCCEY_07093	2520	188	13.1	Serine carboxypeptidase S28
ANCCEY_01927	2500	2	18.7	heat shock protein hsp20*
ANCCEY_01990	2202	0	18.3	HSP20*
ANCCEY_10340	2200	158	13.0	kelch-like protein involved in protein interactions
ANCCEY_14565	2180	220	47.0	hypothetical unknown nematode protein*Ay-GUT-1
ANCCEY_07160	1946	178	12.2	tetratricopeptide repeat (TPR) mediating interactions
ANCCEY_15648	1924	198	17.9	triacylglycerol lipase
ANCCEY_01992	1851	0	18.2	HSP20*
ANCCEY_12597	1827	240	22.6	Retinoid dehydrogenases
ANCCEY_07094	1769	120	56.4	two domains Serine carboxypeptidase S28;
ANCCEY_12249	1687	164	20.1	zinc finger protein,regulating the immune response
ANCCEY_01029	1676	130	37.0	SCP / Tpx-1 / Ag5 / PR-1
ANCCEY_03232	1542	4	42.2	membrane chitin binding Peritrophin-A domain
ANCCEY_05263	1460	109	77.1	Nematode specific protein with unknown*Ay-GUT-1
ANCCEY_10709	1453	0	43.6	C-type lectin *
ANCCEY_00663	1450	6	33.6	cold-shock DNA-binding domain protein
ANCCEY_06153	1402	0	43.5	fibrous sheath CABYR binding (FSCB), protein
ANCCEY_10726	1388	23	20.6	ankyrin repeat and zinc finger domain protein
ANCCEY_08571	1375	87	55.9	two-domain SCP / Tpx-1 / Ag5 / PR-1
ANCCEY_13713	1372	0	17.4	Chondroitin proteoglycan, structural components
ANCCEY_02218	1304	90	26.6	SCP / Tpx-1 / Ag5 / PR-1
ANCCEY_10463	1198	15	20.7	HMG-box protein, involving in DNA binding
ANCCEY_14467	1165	14	16.2	platelet inhibitor
ANCCEY_09034	1113	132	19.1	hypothetical unknown nematode protein
ANCCEY_10803	1110	144	35.6	Organic solute transporter transmembrane protein
ANCCEY_00957	1087	37	44.2	Pepsin-like aspartic proteases
ANCCEY_10074	1029	49	61.0	NRAMP ion transporter

ANCCEY_12936	977	93	20.2	Papain family cysteine protease (cathepsin B)
ANCCEY_11119	917	92	35.8	two-domain SCP / Tpx-1 / Ag5 / PR-1
ANCCEY_07096	899	93	216.8	thymus-specific serine protease
ANCCEY_12135	892	97	16.3	Zinc finger domain containing protein
ANCCEY_13714	859	4	21.7	Chondroitin proteoglycan, structural components
ANCCEY_05879	852	84	32.1	Pantothenate kinase (PNK)
ANCCEY_05891	847	29	15.6	hypothetical unknown nematode protein
ANCCEY_15683	827	39	16.8	prolipoprotein diacylglyceryl transferase
ANCCEY_09249	823	2	14.3	major sperm protein
ANCCEY_00793	808	50	21.4	Plasma membrane calcium transporter
ANCCEY_15469	787	87	23.2	RNA-binding and processing
ANCCEY_05462	721	5	18.7	Hsp20/alpha crystallin family*
ANCCEY_00467	716	0	33.3	hypothetical unknown nematode protein
ANCCEY_07995	708	80	20.0	Serine/threonine protein kinase signal transduction
ANCCEY_10060	700	72	40.9	Papain family cysteine protease (cathepsin B)*
ANCCEY_06123	682	81	26.9	Fibrillarin-like rRNA methylase
ANCCEY_10570	681	62	28.3	transcription factor
ANCCEY_00548	665	95	24.4	Pur-alpha (PURA)/DNA-binding protein
ANCCEY_06151	664	0	27.2	Chondroitin proteoglycan, structural components
ANCCEY_07258	652	42	12.5	glycolipid transfer protein
ANCCEY_09738	634	47	10.2	hypothetical unknown nematode protein
ANCCEY_07700	631	82	13.5	hypothetical unknown nematode protein
ANCCEY_10863	605	3	18.1	hypothetical unknown nematode protein
ANCCEY_03591	584	66	40.4	ribonucleoside-diphosphate reductase
ANCCEY_14793	568	0	16.4	one-domain SCP / Tpx-1 / Ag5 / PR-1
ANCCEY_06776	555	0	46.8	Zinc finger domain containing protein
ANCCEY_14400	539	35	7.6	hypothetical unknown nematode protein
ANCCEY_02709	535	42	54.5	heme ABC exporter and binding protein as chaperon
ANCCEY_00041	522	1	62.3	Chitin binding Peritrophin-A domain;
ANCCEY_09277	508	1	11.6	homocysteine S-methyltransferase
ANCCEY_08370	503	18	7.2	hypothetical unknown nematode protein
ANCCEY_04125	492	44	105.9	HSP70
ANCCEY_01609	492	53	48.3	RNA-binding protein
ANCCEY_13344	484	19	7.2	Major intrinsic protein (MIP) superfamily
ANCCEY_11325	483	52	72.0	low-density lipoprotein receptor
ANCCEY_10164	471	1	68.0	transporter nucleotide-binding domain
ANCCEY_12199	450	0	20.0	C-type lectin CTL*
ANCCEY_00403	446	46	12.1	GTPase involved in transmitting signals within cells
ANCCEY_04284	446	0	12.2	MSP (Major sperm protein) domain
ANCCEY_01549	442	37 7	28.5	hypothetical unknown nematode protein
ANCCEY_10878	435	27	14.4	Purple acid phosphatases (PAPs)
ANCCEY_05040	415	61	21.7	DnaJ/Hsp40 protein, chaperon
ANCCEY_04371	411	41	30.0	Nematode fatty acid retinoid binding protein
ANCCEY_03696	403	49	31.8	DNA-binding protein
ANCCEY_05067	396	0	28.2	Chondroitin proteoglycan
ANCCEY_12393	395	0	14.3	ZYG-11Interactor superfamily for signal conduct
ANCCEY_05381	394	26	20.1	hypothetical unknown nematode protein
ANCCEY_15234	390	42	21.1	Histidine acid phosphatase
ANCCEY_10608	387	41	8.9	Heat shock protein (HSP40)

von Willebrand factor/CTL
SCP / Tpx-1 / Ag5 / PR-1
Transporters
Proteases
Heat shock proteins
*also recognized by immune sera
